# Supplementary material for: Genome-Wide Identification and Functional Characterization Reveals the Pivotal Roles of BnaA8.ATG8F in Salt Stress Tolerance and Nitrogen Limitation Adaptation in Allotetraploid Rapeseed
Source: Int J Mol Sci. 2022 Sep 26;23(19):11318. doi: 10.3390/ijms231911318 (PMC9569553; doi:10.3390/ijms231911318)
Supplement: Supplementary file 1 [file ijms-23-11318-s001.zip › ijms-1913941-Supplementary Figures.pdf]

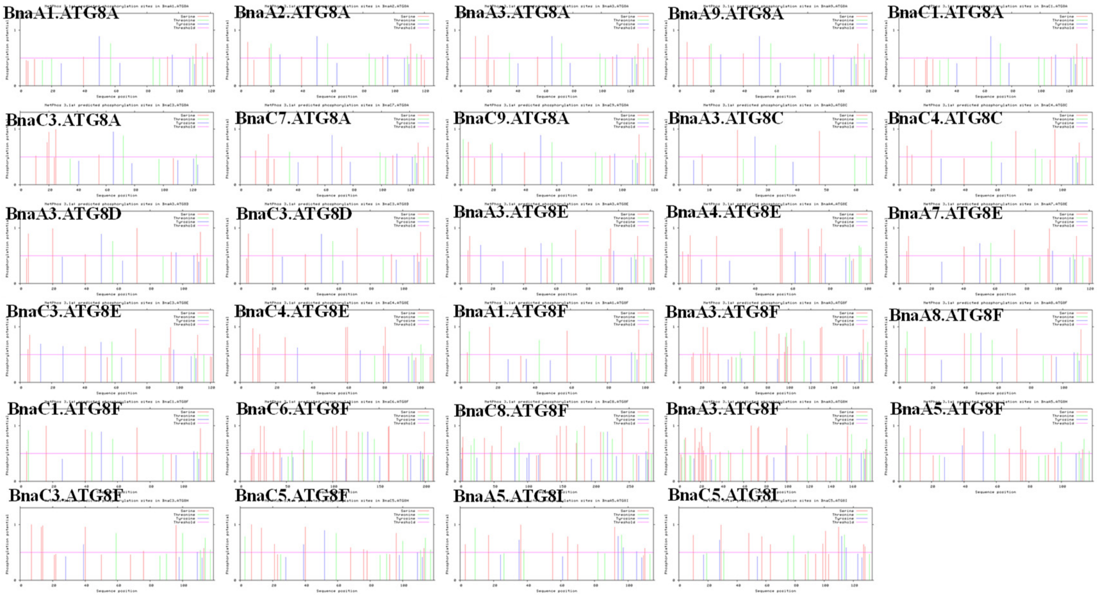

Supplementary Figure S1 Phosphorylation sites of the ATG8 proteins in *Brassica napus*. The NetPhos (http://www.cbs.dtu.dk/services/NetPhos/) 3.1 server was used to predict the presence and location of phosphorylation sites in amino acid sequences of the BnaATG8 proteins.

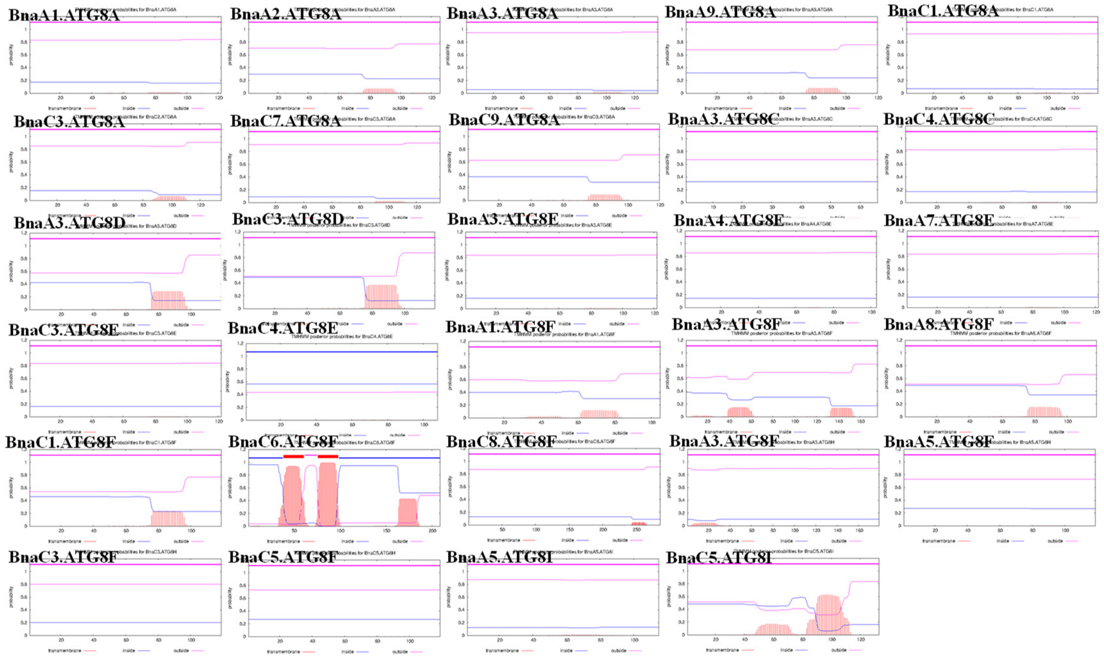

Supplementary Figure S2 Trans-membrane characterization of the ATG8 proteins in *Brassica napus*. The TMHMM (http://www.cbs.dtu.dk/services/TMHMM/) tool was used to predict the transmembrane topology of the BnaATG8 proteins.

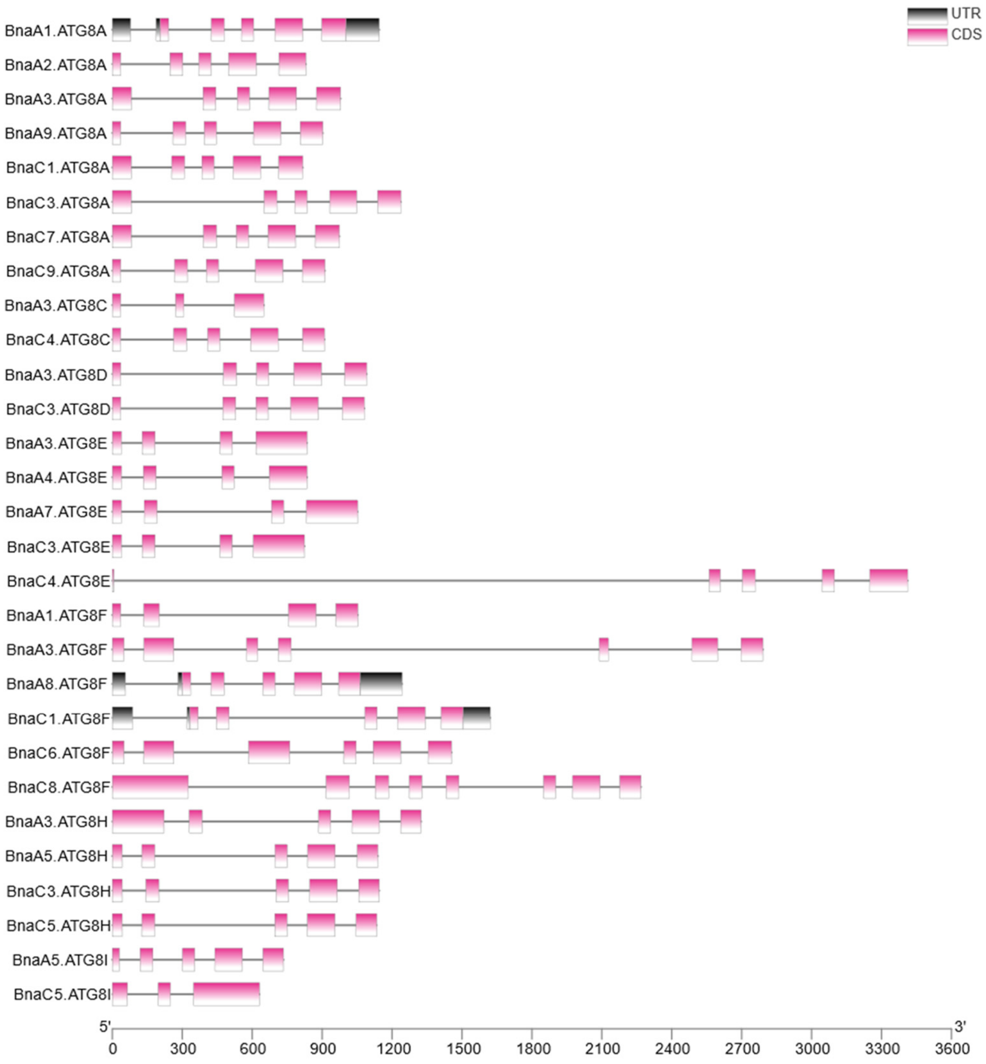

Supplementary Figure S3 Gene structures of the *BnaATG8* family genes in *Brassica napus*. he exon-intron structures of the BnaATG8s were determined by comparing the coding sequence with the corresponding genome sequence. The pink boxes represent exons, black boxes indicate 5′UTR or 3′UTR, and the lines represent introns. The diagram was obtained using the TBtools.

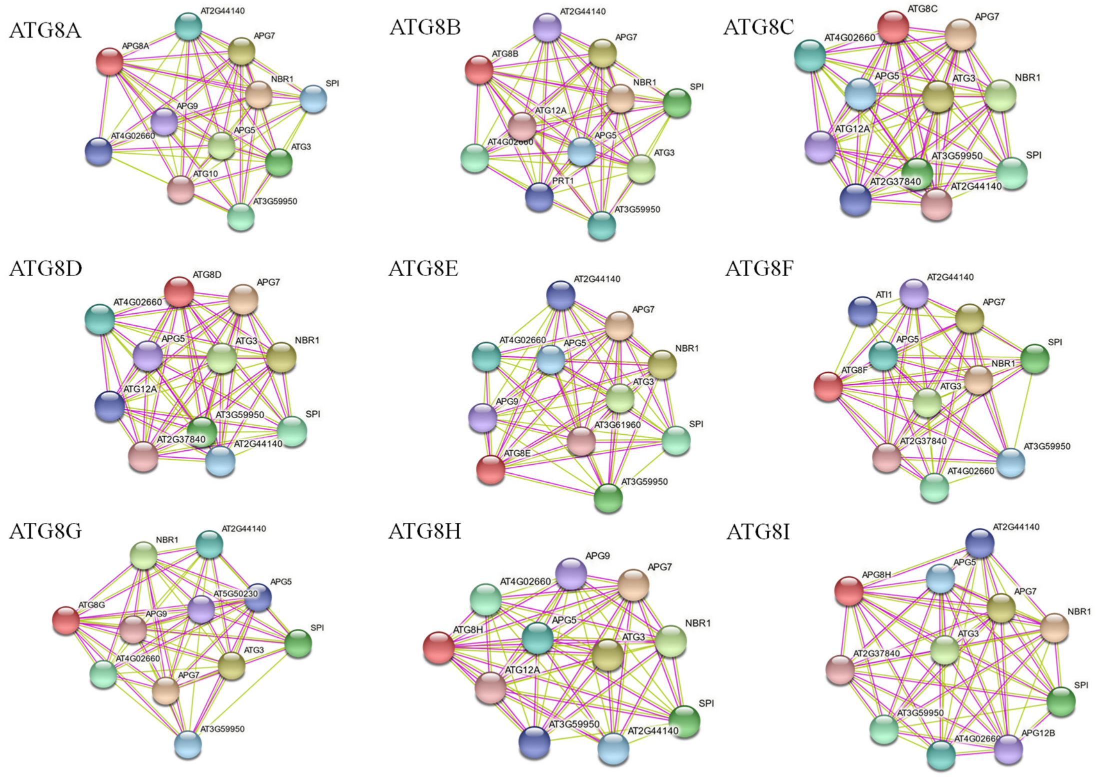

Supplementary Figure S4. Protein–protein interaction networks involving the ATG8s. The interaction networks of ATG8A (A), ATG8B (B), ATG8C (C), ATG8D (D), ATG8E (E), ATG8F (F), ATG8G (G), ATG8H (H) and ATG8I (I)with other proteins were constructed by the STRING web-server.

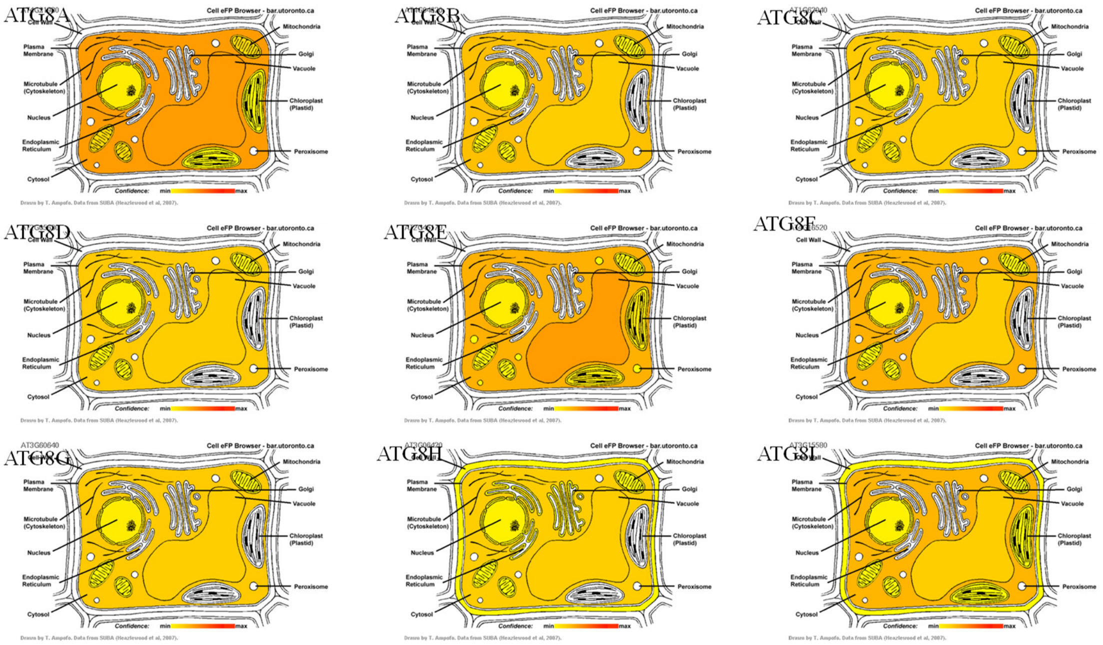

Supplementary Figure S5 Cell-specific expression patterns of the *ATG8* genes in *Arabidopsis thaliana*. Relative expression abundances of *ATG8a-f.* The red and yellow color indicates relative high and low expression levels of AtATG8s.

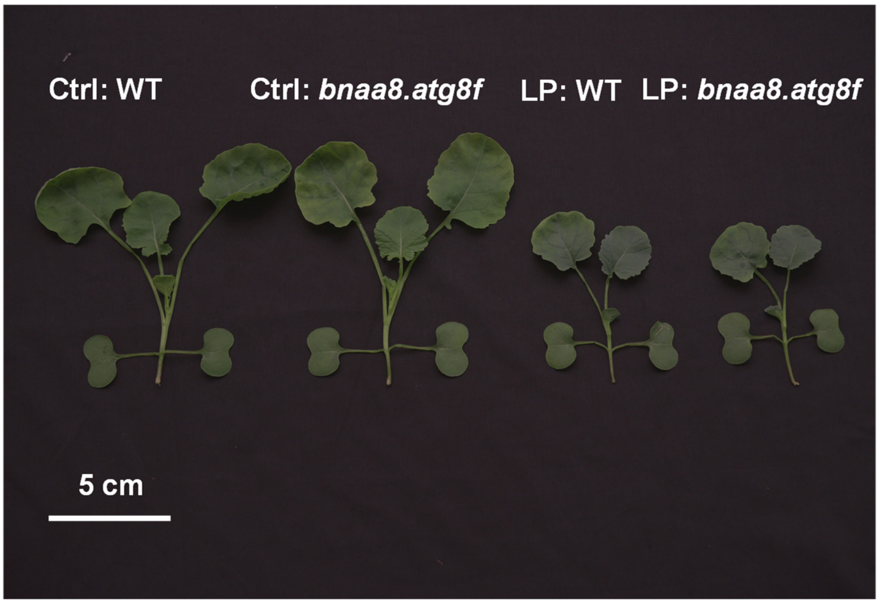

Supplementary Figure S6 Identification of morphological, physiological and gene expression levels of WT and *bnaa8.atg8f* under low phosphorus. Representative images of leaves of the WT and *bnaa8.atg8f* after 5 d treatment with 5 μM KH_2PO_4 and 250 μM KH_2PO_4 for 5 d. Ctrl, control.

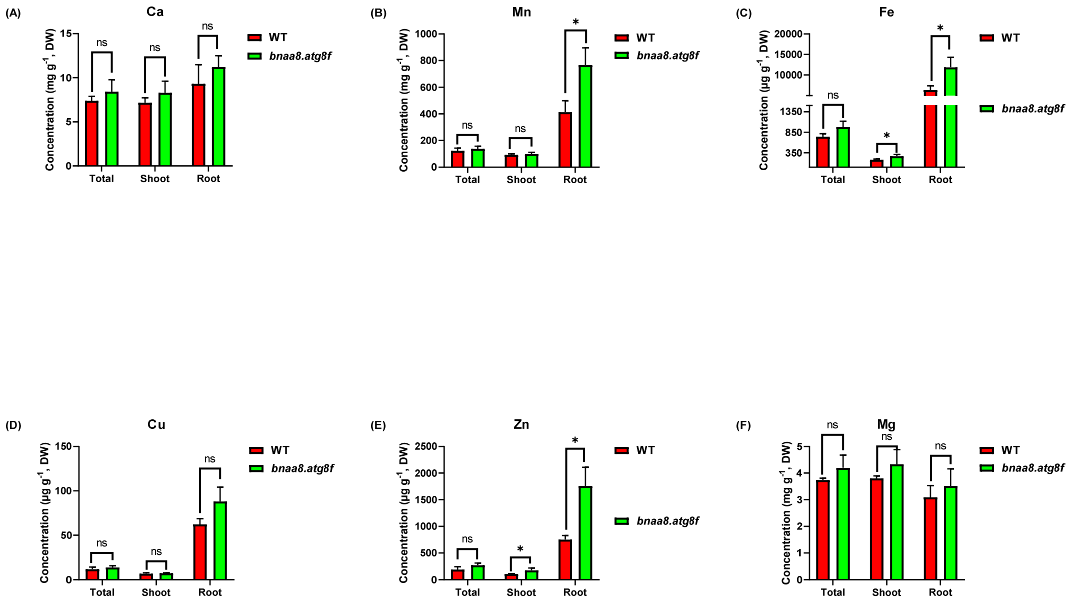

Supplementary Figure S7 Cation profiling of WT and *bnaa8.atg8f* under salinity condition**.** Concentrations of calcium (Ca, A), magnesium (Mn, B), iron (Fe, C), manganese (Mn, D), copper (Cu, E), and zinc (Zn, E) in the whole plants, shoots, and roots. Data are means (±SD), n=5. Significant differences were determined using Student's t-test: *p < 0.05, **p < 0.01; ***p < 0.001; ns, not significant.

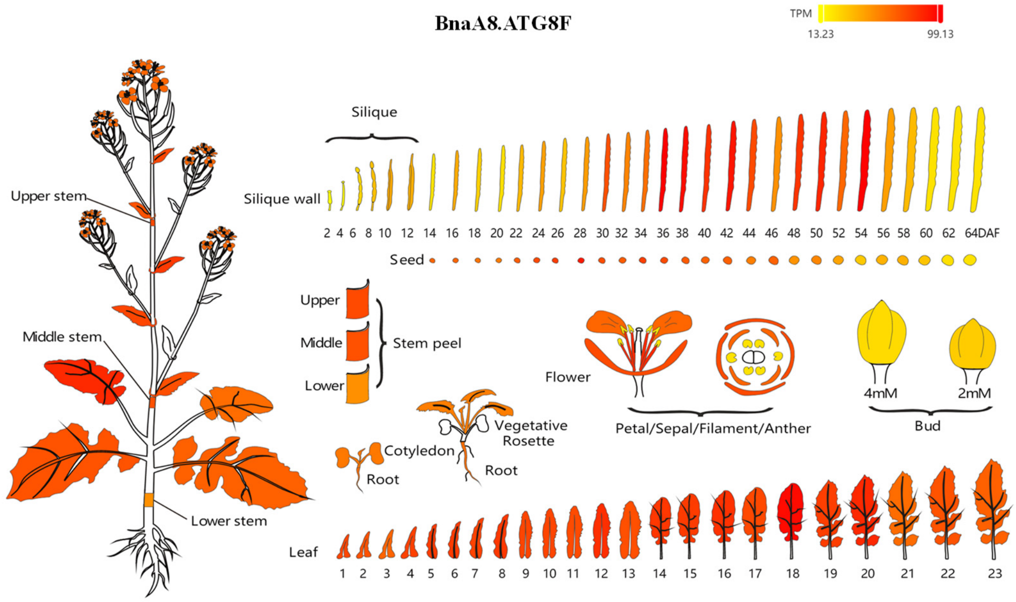

Supplementary Figure S8 Tissue-specificity expression analysis of *BnaA8.ATG8F* in rapeseed plants at the whole growth stages. The expression profiling of target genes was retrieved from the *Brassica napus* transcriptome information resource (BnTIR) database (http://yanglab.hzau.edu.cn/BnTIR).

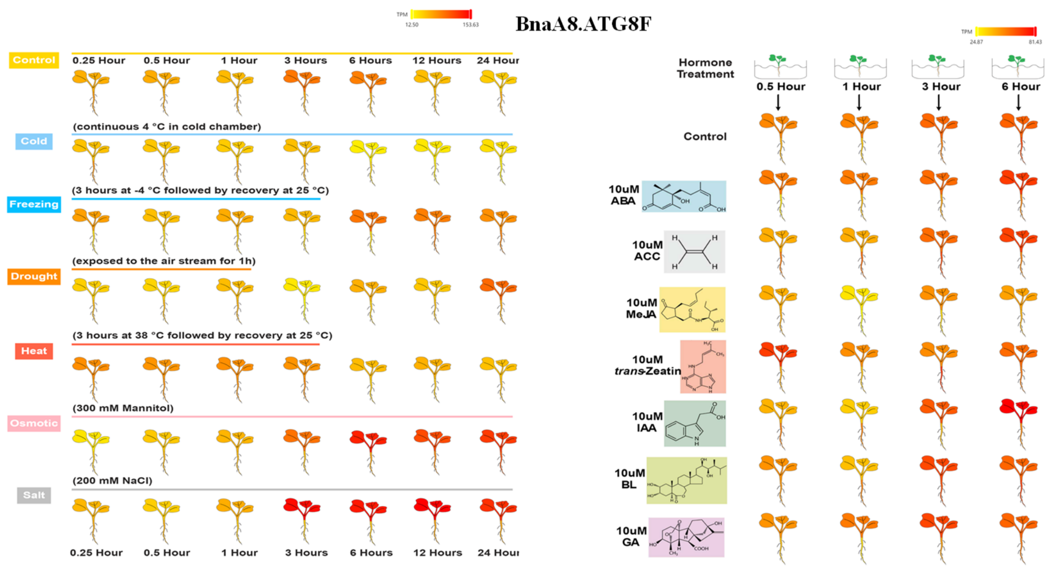

Supplementary Figure S9 Transcriptional level analysis of *BnaA8ATG8F* in response to abiotic stress (0-24 h) and hormones (0-6 h) in rapeseed. The expression profiling of target genes was retrieved from the *Brassica napus* transcriptome information resource (BnTIR) database (http://yanglab.hzau.edu.cn/BnTIR).
