# Supplementary material for: Genome-Wide Identification and Functional Characterization Reveals the Pivotal Roles of BnaA8.ATG8F in Salt Stress Tolerance and Nitrogen Limitation Adaptation in Allotetraploid Rapeseed
Source: Int J Mol Sci. 2022 Sep 26;23(19):11318. doi: 10.3390/ijms231911318 (PMC9569553; doi:10.3390/ijms231911318)
Supplement: Supplementary file 1 [file ijms-23-11318-s001.zip › ijms-1913941-Supplementary Tables.pdf]

Table S1. Copy number of *ATG8* genes in *Arabidopsis* and *Brassica* crops.

Gene name	***Arabidopsis thaliana*** **(125 Mb)**	***Brassica rapa*** **(465 Mb)**	***Brassica oleracea*** **(485 Mb)**	***Brassica napus*** **(1,130 Mb)**
ATG8A	1	4	6	8
ATG8B	1	0	1	0
ATG8C	1	2	2	2
ATG8D	1	0	0	2
ATG8E	1	2	2	5
ATG8F	1	2	3	6
ATG8G	1	0	0	0
ATG8H	1	2	2	4
ATG8I	1	1	1	2
Total	9	13	17	29

Table S2. Molecular characterization of autophagy-related 8 genes in *Arabidopsis* and rapeseed

Gene ID	Gene name	Chromosome	MW (kDa)	PI	II	AI	GRAVY	Subcellular localization
AT4G21980	*AtATG8A*		13.69	8.78	39.51	85.49	-0.24	cyto
BnaA01T0040000WE	*BnaA1.ATG8A*	chrA1	13.78	6.59	32.1	84.67	-0.23	cyto
BnaA02T0252700WE	*BnaA2.ATG8A*	chrA2	14.12	8.78	37.87	86.67	-0.25	cyto
BnaA03T0470100WE	*BnaA3.ATG8A*	chrA3	15.46	8.61	36.32	84.01	-0.08	cyto
BnaA09T0224000WE	*BnaA9.ATG8A*	chrA9	13.64	7.78	40.87	83.67	-0.31	cyto
BnaC01T0120100WE	*BnaC1.ATG8A*	chrC1	15.48	6.59	35.15	86.13	-0.12	cyto
BnaC03T0624600WE	*BnaC2.ATG8A*	chrC2	15.48	9.06	38.95	83.85	-0.25	cyto
BnaC07T0384500WE	*BnaC3.ATG8A*	chrC3	15.42	8.95	34.19	84.74	-0.14	cyto
BnaC09T0256400WE	*BnaC9.ATG8A*	chrC9	13.65	7.78	36.18	86.08	-0.32	cyto
AT4G04620	*AtATG8B*		13.88	8.61	37.83	79.1	-0.29	cyto
AT1G62040	*AtATG8C*		13.73	8.83	49.75	85.97	-0.39	cyto
BnaA03T0589400WE	*BnaA3.ATG8C*	chrA3	7.70	9.45	32.78	90	-0.38	cyto
BnaC04T0234400WE	*BnaC4.ATG8C*	chrA4	13.62	7.92	46.54	89.24	-0.29	cyto
AT2G05630	*AtATG8D*		13.91	7.93	45.72	88.58	-0.29	cyto
BnaA03T0399000WE	*BnaA3.ATG8D*	chrA3	13.72	7.9	50.46	88.49	-0.33	cyto
BnaC03T0408800WE	*BnaC3.ATG8D*	chrC3	13.71	7.9	50.34	88.49	-0.33	cyto
AT2G45170	*AtATG8E*		13.95	7.82	31.25	83.03	-0.42	nucl
BnaA03T0231500WE	*BnaA3.ATG8E*	chrA3	13.93	8.69	29.43	83.85	-0.52	nucl
BnaA04T0272200WE	*BnaA4.ATG8E*	chrA4	11.74	5.46	40.75	94.66	-0.42	nucl
BnaA07T0195100WE	*BnaA7.ATG8E*	chrA7	13.87	6.33	26.79	87.05	-0.41	nucl
BnaC03T0195700WE	*BnaC3.ATG8E*	chrC3	13.91	8.69	30.28	84.67	-0.49	nucl
BnaC04T0588400WE	*BnaC4.ATG8E*	chrC4	12.41	5.5	41.81	84.81	-0.54	nucl
AT4G16520	*AtATG8F*		13.76	8.73	35.08	83.8	-0.41	cyto
BnaA01T0105500WE	*BnaA1.ATG8F*	chrA1	11.79	5.4	27.59	86.38	-0.24	cyto
BnaA03T0443400WE	*BnaA3.ATG8F*	chrA3	19.96	7.73	46.58	83.64	-0.28	cyto
BnaA08T0093200WE	*BnaA8.ATG8F*	chrA8	13.52	7.84	29.97	85.93	-0.18	cyto
BnaC01T0182300WE	*BnaC1.ATG8F*	chrC1	13.50	6.6	27.63	84.37	-0.43	cyto
BnaC06T0196800WE	*BnaC6.ATG8F*	chrC6	23.61	6.84	40.52	96.03	0.05	cyto
BnaC08T0097700WE	*BnaC8.ATG8F*	chrC8	33.42	5.2	45.02	91.29	-0.34	cyto
AT3G60640	*AtATG8G*		13.94	5.87	42.24	79.67	-0.45	cyto
AT3G06420	*AtATG8H*		13.88	6.83	41.1	72.86	-0.45	nucl
BnaA03T0314100WE	*BnaA3.ATG8H*	chrA3	20.49	9.23	42.55	81.62	-0.23	nucl
BnaA05T0417300WE	*BnaA5.ATG8H*	chrA5	13.71	7.85	40.44	68.74	-0.46	nucl
BnaC03T0296700WE	*BnaC3.ATG8H*	chrC3	13.74	6.09	39.37	76.13	-0.36	nucl
BnaC05T0512100WE	*BnaC5.ATG8H*	chrC5	13.70	6.9	39.4	68.74	-0.44	nucl
AT3G15580	*AtATG8I*		13.25	7.7	44.02	82.26	-0.23	cyto
BnaA05T0347900WE	*BnaA5.ATG8I*	chrA5	13.23	6.59	47.23	86.52	-0.21	cyto
BnaC05T0420900WE	*BnaC5.ATG8I*	chrC5	14.98	8.5	37.98	96.09	-0.15	cyto

Note: GRAVY, grand average of hydropathy; II, instability index; MW, molecular weight; pI, isoelectric point; PM, plasma membrane; TM, transmembrane; Vac, vacuole; Endo, endosome

Table S3. Molecular characterization of autophagy-related 8 genes in *Arabidopsis* and rapeseed.

Gene ID	Gene name	Block	Protein Length (aa)	CDS Length (bp)	Exon/intron	Ka	Ks	Ka/Ks	Divergent time (Mya)
AT4G21980	*AtATG8A*	U	122	369					
BnaA01T0040000WE	*BnaA1.ATG8A*	U	122	369	5	0.037	0.432	0.085	14.38
BnaA02T0252700WE	*BnaA2.ATG8A*	U	126	381	5	0.054	0.532	0.101	17.72
BnaA03T0470100WE	*BnaA3.ATG8A*	U	137	414	5	0.036	0.412	0.089	13.73
BnaA09T0224000WE	*BnaA9.ATG8A*	U	120	363	5	0.058	0.555	0.105	18.49
BnaC01T0120100WE	*BnaC1.ATG8A*	U	137	414	5	0.040	0.430	0.094	14.35
BnaC03T0624600WE	*BnaC3.ATG8A*	U	135	408	5	0.040	0.390	0.103	13.00
BnaC07T0384500WE	*BnaC7.ATG8A*	U	139	420	5	0.109	0.479	0.227	15.96
BnaC09T0256400WE	*BnaC9.ATG8A*	U	120	363	5	0.066	0.555	0.119	18.49
AT4G04620	*AtATG8B*	O	122	369					
AT1G62040	*AtATG8C*	D	119	360					
BnaA03T0589400WE	*BnaA3.ATG8C*	D	66	201	3	0.077	0.340	0.226	11.32
BnaC04T0234400WE	*BnaC4.ATG8C*	D	119	360	5	0.049	0.231	0.211	7.69
AT2G05630	*AtATG8D*	G	120	363					
BnaA03T0399000WE	*BnaA3.ATG8D*	G	119	360	5	0.015	0.531	0.027	17.70
BnaC03T0408800WE	*BnaC3.ATG8D*	G	119	360	5	0.018	0.389	0.047	12.98
AT2G45170	*AtATG8E*	J	122	369					
BnaA03T0231500WE	*BnaA3.ATG8E*	J	122	369	4	0.030	0.433	0.070	14.45
BnaA04T0272200WE	*BnaA4.ATG8E*	J	103	312	4	0.118	0.486	0.242	16.21
BnaA07T0195100WE	*BnaA7.ATG8E*	J	122	369	4	0.037	0.397	0.093	13.24
BnaC03T0195700WE	*BnaC3.ATG8E*	J	122	369	4	0.034	0.482	0.071	16.06
BnaC04T0588400WE	*BnaC4.ATG8E*	J	108	327	5	0.096	0.480	0.200	16.01

AT4G16520	*AtATG8F*	U	121	366					
BnaA01T0105500WE	*BnaA1.ATG8F*	U	105	318	4	0.067	0.373	0.180	12.43
BnaA03T0443400WE	*BnaA3.ATG8F*	U	176	531	7	0.152	0.438	0.348	14.58
BnaA08T0093200WE	*BnaA8.ATG8F*	U	118	357	5	0.030	0.234	0.127	7.81
BnaC01T0182300WE	*BnaC1.ATG8F*	U	119	360	5	0.022	0.394	0.056	13.15
BnaC06T0196800WE	*BnaC6.ATG8F*	U	209	630	6	0.040	0.370	0.109	12.33
BnaC08T0097700WE	*BnaC8.ATG8F*	U	286	861	8	0.030	0.252	0.118	8.39
AT3G60640	*AtATG8G*	N	121	366					
AT3G06420	*AtATG8H*	F	119	360					
BnaA03T0314100WE	*BnaA3.ATG8H*	F	179	540	5	0.052	0.531	0.097	17.71
BnaA05T0417300WE	*BnaA5.ATG8H*	F	119	360	5	0.069	0.373	0.185	12.45
BnaC03T0296700WE	*BnaC3.ATG8H*	F	119	360	5	0.050	0.467	0.106	15.56
BnaC05T0512100WE	*BnaC5.ATG8H*	F	119	360	5	0.065	0.373	0.174	12.45
AT3G15580	*AtATG8I*	F	115	348					
BnaA05T0347900WE	*BnaA5.ATG8I*	F	115	348	5	0.027	0.349	0.077	11.63
BnaC05T0420900WE	*BnaC5.ATG8I*	*F*	133	402	3	0.051	0.449	0.113	14.957

Note: CDS, coding sequence; Ka, non-synonymous nucleotide substitution rate; Ks, synonymous nucleotide substitution rate.

Table S4. Gene used to validate the transcriptome information and sequence of primers

gnen	F sense primer	R anti-sense primer
BnaA8.ATG8F	5-TGCTCGGATCAGAGAGAAGTA-3	5-TGCCCCACTGTCAAATCAGCC-3
BnaC8.NRT2.1a	5-AAAGGTACTGAGGAGCACTATTATGG-3	5-GTATTCTGAGGCGGCGTAGC-3
BnaC2.NAR2.1	5-TCAAGAAGCTCCTTTTCGCG-3	5-TAAATTCAGGCTCCTTTGTAGCC-3
BnaC2.NRT1.7	5-GCTGGATCATCGGCTTTAGC -3	5-GAGCTTCATCTTTCGCTTCTTG -3
BnaA3.NRT1.8	5-GGGTATGGTGGTTATCAGCCC -3	5-CGAAAGGAGCGATCCGAGG -3
BnaEF1-α	5-GCCTGGTATGGTTGTGACCT -3	5- GAAGTTAGCAGCACCCTTGG-3
BnaTublin	5-CAGCAATACAGTGCCTTGAGTG-3	5-CCTGTGTACCAATGAAGGAAAGCC -3
